# Diversity, local knowledge and use of stingless bees (*Apidae: Meliponini*) in the municipality of Nocupétaro, Michoacan, Mexico

**DOI:** 10.1186/1746-4269-10-47

**Published:** 2014-06-05

**Authors:** Alejandro Reyes-González, Andrés Camou-Guerrero, Octavio Reyes-Salas, Arturo Argueta, Alejandro Casas

**Affiliations:** 1 Centro de Investigaciones en Geografía Ambiental, Universidad Nacional Autónoma de México, Morelia, Mexico; 2 Centro de Investigaciones en Ecosistemas (CIEco), Universidad Nacional Autónoma de México, Morelia, Mexico; 3 Escuela Nacional de Estudios Superiores Unidad Morelia, Universidad Nacional Autónoma de México, Morelia, Mexico; 4 Facultad de Química, Universidad Nacional Autónoma de México, Mexico City, Mexico; 5 Centro Regional de Investigaciones Multidisciplinarias, Universidad Nacional Autónoma de México, Cuernavaca, Mexico

**Keywords:** Stingless bees, Ethnoentomology, Balsas River Basin

## Abstract

**Background:**

Stingless bees were significant resources managed by Mesoamerican peoples during pre-Columbian times and remain important in particular areas. Our study aimed at inventorying stingless bees’ species, traditional knowledge and forms of use and management of them at the municipality of Nocupetaro, Michoacán, Mexico, a region of the Balsas River Basin.

**Methods:**

We inventoried the stingless bees of the municipality of Nocupétaro, Michoacán, México, through extensive collecting of bee specimens in different vegetation types. We then conducted semi-structured interviews to local experts in order to document their knowledge and management techniques of stingless bees’ species.

**Results:**

We identified a total of eight stingless bees’ species in the study area as well as three additional unidentified taxa recognized by people through the local names. Our inventory included one new record of species for the region (*Lestrimelitta chamelensis* Ayala, 1999). The taxa identified are all used by local people. *Scaptotrigona hellwegeri* Friese, 1900; *Melipona fasciata* Latreille, 1811; *Frieseomelitta nigra* Cresson, 1878 and *Geotrigona acapulconis* Strand, 1919 are particularly valued as food (honey), medicinal (honey and pollen), and material for handcrafts (wax). All species recorded are wild and their products are obtained through gathering. On average, local experts were able to collect 4 nests of stingless bees per year obtaining on average 6 L of honey and 4 Kg of wax but some came to collect up 10–12 hives per year (18 L of honey and 24 Kg of wax).

**Conclusions:**

Local knowledge about use, management and ecological issues on stingless bees is persistent and deep in the study area. Information about this group of bees is progressively scarcer in Mexico and significant effort should be done from ethnobiological and ecological perspectives in order to complement the national inventory of bee resources and traditional knowledge and management of them.

## Background

Stingless bees (*Apidae: Meliponini*) are widely distributed in tropical and subtropical regions of the world [1]. Nearly 500 species have been described, most of them in the tropics of the New World, which is considered the main area of diversification of this group of organisms [1]. A total of 46 species of 11 genera have been recorded in Mexico, 12 of them (nearly 26%) being endemic to the territory of this country [2,3]. Stingless bees are mainly associated to tropical dry and humid forests, in low and warm lands although some species can be found in cloud forest and pine-oak forests in highlands [2]. Particularly in the Central-western region of Mexico a high diversity and endemism of stingless bees’ species has been recorded particularily in three main zones: the Pacific coast, the mountainous area of the Sierra Madre del Sur and the Balsas River Basin [2]. However, relatively few studies on diversity and ecological aspects of the *Meliponini* bees are still available, mainly in the case of the Balsas River in Michoacan.

Stingless bees are organisms of high ecological importance. It is known that between 30 to 50% of flowering plant species of tropical areas of the New World are pollinated by bees a high proportion of them belonging to the taxonomic group analyzed in this study [4,5]. This fact and the current global crisis of pollinators [6] caused by human activities impacting their diversity and abundance [7-9] are priority issues for both science and society.

Stingless bees have been relevant in human culture. They have provided resources and have been part of the social and religious life of several peoples that have developed management techniques of these insects which are technically called “meliponiculture” [10]. In the tropical Americas this activity originated in pre-Columbian times, with particularly important advances in Mesoamerica, the cultural area comprised between southern Mexico and North-eastern Costa Rica, as well as in the Andean region [10,11].

The traditional Mesoamerican meliponiculture aims to provide honey (which was an important food and medicine), and these products played an important role both economically and sacred [12]. During pre-Columbian times in Mexico four main zones are recognized to have been areas where stingless bees were raised: 1) The Yucatán Peninsula, 2) the Coast of the Gulf of Mexico, 3) The Pacific Coast between the current states of Jalisco and Sinaloa, and 4) the Balsas River Basin, mainly in the current states of Guerrero and Michoacán [13-16]. In these areas important management practices were developed in relation to extraction of products from natural areas, as well as breeding of bees in artificial nests constructed with various techniques.

At present, the actual practices of the Mesoamerican meliponiculture must be considered as a strong and persistent culture of the indigenous and peasant peoples. Presence of old management techniques have been documented to currently occur among the Maya of the Yucatán Peninsula [17], the Nahuas and Totonac of the Sierra Norte in the state of Puebla, in southern Veracruz (Popolucas) [18] and in the Isthmus of Tehuantepec (Zapotec, Mixe, Zoque, Nahuas y Popolucas) [19]. According to González-Acereto [17] in the Yucatan peninsula and southern Mexico the traditional use of several species of stingless bees still persists, where the most representative is “*xunan-cab*” (*Melipona beecheii*) being the most important cultivated species whose honey and pollen are used in traditional medical and regionally marketed. The Náhuatl and Totonac peoples of the Sierra Norte manage the “*pisil-nekmej*” (*Scaptotrigona mexicana*) which has an important regional marketing because of its medicinal honey [18]. In these regions civil organizations and academic institutions have collaborated in documenting, rescuing traditional meliponiculture and innovating them with current techniques and materials. Particularly important are also the experiences of innovation techniques in the southeastern states of Tabasco and Chiapas [20], as well as areas of the Coast of the state of Guerrero where two species (*Melipona fasciata* and *Scaptotrigona hellwegeri*) have been recorded to be managed by Nahuas people in a village of Atoyac de Alavarez [21]. According to Ayala [3], of the 46 species of stingless bees described for Mexico, 19 of them are currently used today for crop pollination, honey (food and medicine), pollen and cerumen (crafts and folk art).

Although recognized as an important region in terms of stingless bees’ diversity and culture, the Balsas River Basin is currently particularly poor in information on species richness, traditional knowledge and quality of their honey. We expect to find in the study area traditional ecological knowledge (TEK), management techniques, as well as high species richness of stingless bees, and we started to document such a supposition in part of this vast area. In order to fill the void contribute to this effort, our study was directed to inventorying the species richness of stingless bees in the region of the Balsas River Basin in the state of Michoacán, documenting local knowledge and management techniques by people of communities in the municipality of Nocupétaro. We consider this relevant, in view of the conservation of stingless bees’ species while exploring the potential of alternative productive projects.

## Methods

### Study area

**Figure 1:**
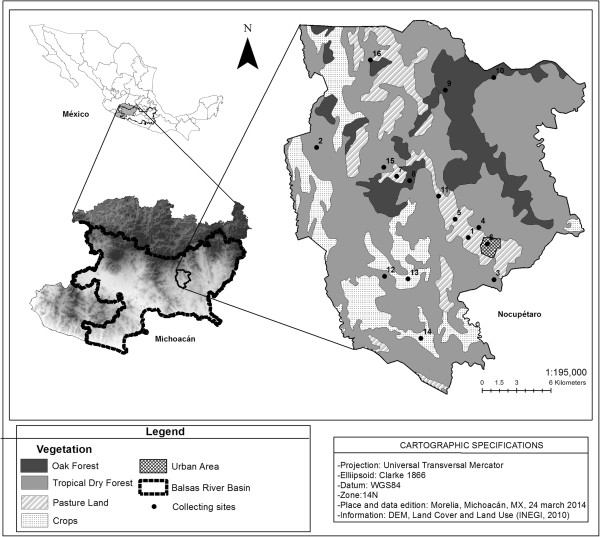
**Map of the study area.** The municipality of Nocupétaro and the different types of vegetation. The category of oak forest includes oak – pine mixed forest.

Our study area included the territory of communities belonging to the municipality of Nocupétaro, Michoacán, located in the Central-western region of Mexico within the Balsas River Basin (Figure 1). Nocupétaro comprises elevations between 500 to 1,800 m, the climate is warm sub-humid characterized by summer rainfall ranging 700–1,100 mm, and temperatures between 20 and 28°C [22]. Vegetation is mainly tropical dry forest in the lowlands (56.6%) (Figure 1), followed by induced pasture lands (18.5%), oak forest (8.0%), crops (7.0%) and mixed oak-pine forest (9.5%) [23]. Nocupétaro means *lugar en el valle* (place in the valley in Phurépecha language), and is now a mestizo municipality with a total population of 7,799 inhabitants [24]. The main economic activities are agriculture (corn, peanuts and sesame) and livestock (cattle, goats and poultry) [22].

### Entomological and ethnoentomological inventory

We inventoried the apifauna centering our attention on stingless bees. For such a purpose we conducted extensive collecting of bee specimens through the method of “direct searching” [25] in 16 sites (Table 1) located in the vegetation types of: a) tropical dry forest, b) pasture lands, c) oak forest, d) maize crops and e) urban areas. We also conducted directed collecting with the help of local experts of the villages studied who have extensive knowledge of the stingless bees in the study area. For capturing bees we used entomological nets 18 inches diameter, collecting for 12 hours per site; also we used Malaise traps which were established for 28 days in the most inaccessible places (2 sites) where local experts knew about the presence of bees.

**Table 1:** Collecting sites and their biophysical characteristics

**Collecting site**	**Locality**	**Altitude (m)**	**Landform**	**Vegetation**
1	Nocupétaro	660	Flood-plain	Crops
2	Atravesaño	858	Hillslope	Tropical Dry Forest
3	Agua Zarca	652	Hillslope	Tropical Dry Forest
4	La Minita	691	Foothill	Pastureland
5	El Llano	634	Plain	Crops
6	Nocupétaro	650	Plain	Urban Area
7	Zapotito	888	Hill	Pastureland
8	Mariana	879	Plain	Oak forest
9	Cuispio	1040	Mountain	Pastureland/Oak forest
10	El Platanal	1210	Mountain	Oak forest
11	Ceiba Prieta	667	Flood-plain	Pastureland
12	Las Pilas	663	Plain	Tropical Dry Forest
13	Santo Domingo	603	Hill	Crops
14	Estancia Grande	555	Plain	Crops
15	Sauz	956	Hillslope	Tropical Dry Forest
16	Loma del Copal	1400	Mountain	Pastureland/Oak forest

The specimens collected were slaughtered in lethal chambers with ethyl-acetate [26] for subsequent mounting. These specimens were deposited in entomological boxes for taxonomic identification, which was performed using taxonomic dichotomous keys [1,2], and with the advice of specialists from the Universidad Michoacana de San Nicolas de Hidalgo (UMSNH), Morelia, Michoacan, Mexico. The nomenclature of the species recorded was done according to Camargo and Pedro [27].

The specimens collected were deposited in the Entomological Collection of the Museum of Zoology, Faculty of Sciences, UNAM, Mexico. In addition to the taxonomic identification of specimens collected, distribution (defined as the proportion of sites where species were collected) was recorded.

### Traditional knowledge and management techniques

For documenting traditional knowledge, use forms and management techniques by local people on stingless bees, we carried out 20 semi-structured interviews to local experts in 18 villages of the Nocupétaro municipality (representing 15% of the total localities and the diversity of vegetation types). The interviews were conducted using the collected specimens, in order to confirm the information. These local experts are locally recognized as *colmeneros* who are skilled in collecting stingless bees’ products. Interviews were divided into two main sections: a) local knowledge of stingless bees (including names, spatial and temporal distribution, abundance, feeding and nesting characteristics of stingless bees), and b) use and management (including management techniques, forms of use and preparation of products obtained from stingless bees, the division of labor in obtaining and preparation of such products, productivity and preference of usage of the different species of stingless bees).

In addition, we used the method of “participant observation” [28,29] working with 8 local experts of the study area, by recording the activities of extraction of products from the beehive and the search for nests in the wild. The group of local experts integrated in the study was formed by 19 men and 1 woman of 55 years on average. Local experts were contacted through the snowball sampling method [30,31], which involved the construction of a network of experts based on the recommendation of the experts themselves. Finally, we defined a mention index (MI) (proportion of bee’s species mentions divided by the total number of interviews) as a way to recognize the stingless bees locally best known and the preferences of local use [32].

## Results

### Richness of stingless bees in Nocupétaro

We recorded a total of eight species of stingless bees belonging to six genera (Table 2). *Frieseomelitta nigra* Cresson, 1878 was the species most widely found, appearing in 12 of the 16 collecting sites (75%) and covering the entire spectrum of vegetation types where the collections were conducted (Table 2). At a second level *Scaptotrigona hellwegeri* Friese, 1900 was found in 10 sites (62.5%) in all the vegetation types and *Geotrigona acapulconis* Strand, 1919 at 8 sites (50%) and also found in all the vegetation types (except in the urban area) (Table 2). Moreover, species such as *Trigonisca* sp., were collected at a single site (6.3%), in the case of this species an oak forest at 1210 m. *Lestrimelitta chamelensis* Ayala, 1999 represents a new record for the region and was collected directly in the nest, a hollow log of *cirian* (*Crescentia alata*), in a tropical dry forest at 555 m (Date: 03-07-2010; Collecting method: entomological nets 18 inches diameter; Collector: Alejandro Reyes González; Locality: La Estancia Grande, Nocupétaro, Michoacán 18°58′11, 101°12′49; Identification: Alejandro Reyes González and Aarón Mejía based on [2]). Finally *Melipona fasciata* Latreille, 1811, *Nannotrigona perilampiodes* Cresson, 1878 and *Partamona bilineata* Say, 1837 were collected at 1040 m and 1210 m respectively, in oak forest (Table 2).

**Table 2:** Stingless bees’ species recorded at the municipality of Nocupétaro, Michoacan, Mexico

**Taxon**	**Local name**	**Number of sites/(%)**	**Vegetation types**
1. *Frieseomelitta nigra* Cresson, 1878	Abeja zopilota	12/(75.0)	1,2,3,4,5
2. *Scaptotrigona hellwegeri* Friese, 1900	Abeja bermeja	10/(62.5)	1,2,3,4,5
3. *Geotrigona acapulconis* Strand, 1919	Colmena de tierra/Prieta de tierra	8/(50.0)	1,2,3,5
4. *Trigonisca sp.*	Abeja cepimilla	1/(6.3)	5
5. *Lestrimelitta chamelensis* Ayala, 1999*	Abeja limoncilla	1/(6.3)	3
6. *Melipona fasciata* Latreille, 1811	Colmena real	1/(6.3)	5
7. *Nannotrigona perilampiodes* Cresson, 1878	Abeja trompetera	1/(6.3)	5
8. *Partamona bilineata* Say, 1837	Abeja esculcona	1/(6.3)	5
		n = 16 sites	

*New registration.

Vegetation types: 1) Crops; 2) Pastureland; 3) Tropical dry forest; 4) Urban area; 5) Oak forest.

### Local knowledge on stingless bees

Stingless bees are locally called *colmenas* or *colmenas de palo* (*colmena* is a general term in Spanish used for naming beehives, *colmena de palo* is a woody bee nest); these are bees also called *abejas de monte* (wild bees) or *colmenas que no pican* (stingless bees *colmenas*). The local experts interviewed recognized 11 different types of stingless bees which are identified based on their morphological and behavioral characteristics as summarized in Table 3.

**Table 3:** Local knowledge on stingless bees at the municipality of Nocupetaro, Michoacan

**Local name**	**Taxon**	**Behavior (local knowledge)**	**Morphology (local knowledge)**	**Nesting**	**Use (product)**	**MI (%)**
1. Abeja Bermeja	*Scaptotrigona. hellwegeri*	Defensive (gets tangled in the hair and bites)	Intense reddish median bee	In hollow trunks	H (*m,f*)	90
					B (*m,rm*)	
					P (*m,f*)	
2. A. Cepimilla	*Trigonisca* sp	Bee type that likes people sweat	Very small bee	In trunks, very small nests	H (*m*)	5
3. A. Esculcona	*Partamona. bilineata*	Very defensive (gets tangled in the hair and bites)	Median black Bee	Aerial and exposed nest as termite mound.	B (*rm*)	10
4. A. Limoncilla	*Lestrimelitta. chamelensis*	Docile and attack other bees	Small dark bee, with strong lemon scent	In hollow logs	H (*m*)	35
					B (*rm*)	
5. A. Pintilla	*Unidentified*	Very Defensive (gets tangled in the hair and bites	Similar to *Apis mellifera* in size and color	In hollow logs, cavities between the trunk and the ground	H (*m,f*)	5
					B (*m,rm*)	
					P (*m,f*)	
6. A. Prieta esculcona	*Unidentified*	--	Median and very dark bee	--	B (*rm*)	5
7. A. Sapito	*Unidentified*	Very docile and timid	Small dark bee	--	H (*m,f*)	5
					P (*m,f*)	
8. A. Trompetera	*Nannotrigona perilampiodes*	Very docile and timid	Small bee	In hollow trunks. The nest entrance is shaped trumpet (made of beeswax)	H (*m,f*)	5
					B (*m,rm*)	
					P (*m,f*)	
9. A. Zopilota	*Frieseomelitta nigra*	Docile	Median dark bee, very bright with white wing tips	In hollow logs	H (*m,f*)	90
					B (*rm*)	
					P (*m*)	
10. Colmena real	*Melipona. fasciata*	Very defensive (gets tangled in the hair and bites)	Similar to *Apis mellifera* in size and color	In hollow logs	H (*m,f*)	85
					B (*m,rm*)	
					P (*m,f*)	
11. Colmena de Tierra or Prieta de Tierra	*Geotrigona acapulconis*	Very docile and timid	Median bee completely dark	Buried in the ground	H (*m*)	80
					B (*r*)	

Note. The unidentified stingless bees were referred by local experts but were not collected.

(MI) Mention Index; (H) honey, (B) beeswax, (P) pollen; (*m*) medicinal, (*f*) food, (*rm*) raw material.

Local experts recognized the *abeja bermeja* (*S. hellwegeri*) by its red color and its defensive behavior. The stingless bee called *zopilota* (*F. nigra*) is recognized by its dark color and the wing tips white and it is also characterized by its docile behavior. The *colmena de tierra* or *prieta de tierra* (*G. acapulconis*) is identified by its dark color and its pattern of nesting in the ground as well as being very docile. Moreover, the *colmena real* (*M. fasciata*) is identified by its colors similar to *Apis mellifera*, its hairiness and size. The new record bee *limoncilla* (*L. chamelensis*) which is named for its distinctive lemon scent (typical of the genus *Lestrimellita*) is distinguished by local experts by attacking other species of stingless bees and steal their honey. The *abeja esculcona* (*P. bilneata*) is known for its aerial nesting and its defensive behavior. The bee *cepimilla* (*Trigonisca* sp) is identified by local experts for its small size and its habit of perching on the sweat of the people. The bee *trompetera* (*N. perilampiodes*) nests in hollow trunks and is named this way because the entrance to its nest is shaped like a trumpet made of beeswax. Additionally local experts known and cited three stingless bees’ species that were not collected and identified: *abeja pintilla, abeja prieta esculcona* and *abeja sapito*, (Table 3).

Out of all the species locally recognized, the *abeja bermeja* (*S. hellwegeri*), *abeja zopilota* (*F. nigra*), the *colmena real* (*M. fasciata*), and the *colmena de tierra* (*G. acapulconis*) are the best known, since all of them were referred by more than 80% of the local experts interviewed (Table 3). The *abeja limoncilla* (*L. chamelensis*) is moderately known since 35% of local experts referred to this bee. The least known stingless bees were the *abeja esculcona* (*P. bilneata*), *abeja trompetera* (*N. perilampiodes*) and the *abeja cepimilla* (*Trigonisca* sp).

Local experts expressed a positive perception in relation to stingless bees, all of them said that these bees are “good” since they provide *miel* (honey), *cerumen* (beeswax), and *pasacuareta* (pollen), and they do not cause any damage to people (compared with *Apis mellifera* and wasps). Knowledge of stingless bees is transferred from parents to children and according to the 85% of the local experts interviewed; men know and recognize differences among stingless bees’ species, as well as their behavior and habits patterns, their nesting sites and characteristics, seasons and particular techniques for extracting products. For the contrary, 75% of the local experts interviewed referred to that women know better the techniques used for processing the products, particularly those requiring more specialization such as preparing wax for manufacturing candles, but also the form of preparing pollen and honey for food and medicinal purposes.

### Use of stingless bees’ products

According to 70% of the local experts interviewed, the main motive for using stingless bees has been their wax or *cerumen*, for preparing candles used during the *día de muertos* or *día de las ánimas* in November 2. Beeswax extraction collaterally allowed using honey and pollen. The manufacture of beeswax candles is an activity involving specialized practices which are carried out by women. The first step of preparation of beeswax is its washing with water, then boiling the washed beeswax with water, and repeating the process. The second last cooking without water was the moment for including the candlewick. Beeswax is also used for sealing traditional bowls made of gourds (*Lagenaria siceraria*), and nearly 30% of local experts said that beeswax is commercialized in areas out of Nocupétaro where it is used for managing grafting in fruit trees.

Honey is highly valued for its medicinal and food uses. According to local experts, honey is consumed almost immediately after being extracted, alone or accompanying a hot drink. As medicine, honey of stingless bees is used for treating different illnesses. It is consumed liquid in mixture with different ingredients for treating cold, cough, bronchitis and other respiratory illnesses (with lemon juice, but 15% of local experts interviewed also mentioned agave mescal, ethanol, and fruit pulp of *Crescentia alata*). It is also used as antiseptic, topically put on specific infected parts of the body, particularly eyes and cutaneous injures and infections. As anti-inflammatory honey is used for muscle fatigue, sprains, injures, mauls, dabbed on affected parts. The pollen or “*pasacuareta*” is edible; in general people said that when “*pascuareta*” has yellow colour its consumption should be avoided since it cause vomit.

**Figure 2:**
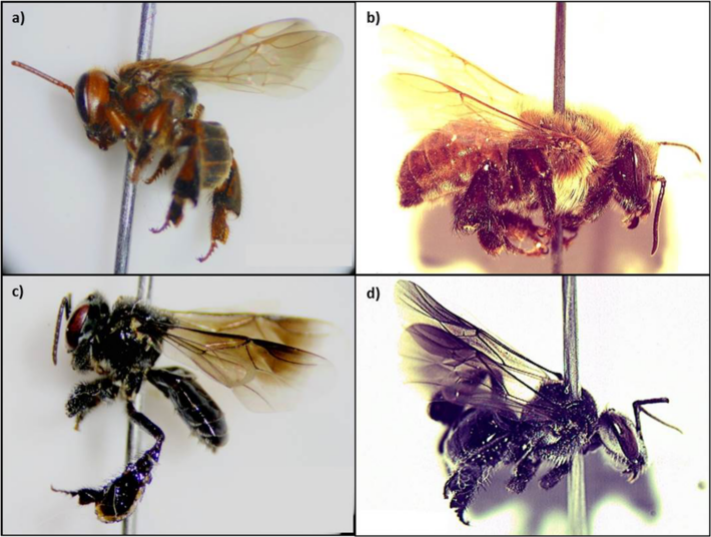
**Stingless bees most mentioned and used in the study area. a)***Scaptotrigona hellwegeri,***b)***Melipona fasciata*, **c)***Frieseomelitta nigra* and **d)***Geotrigona acapulconis.* Photographs: Alejandro Reyes-González.

The use of stingless bees has focused primarily on four species that have been the most widely used, the *abeja bermeja* (*S. hellwegeri*) (MI: 80%), *colmena real* (*M. fasciata*) (MI: 60%), *abeja zopilota* (*F. nigra*) (MI: 55%) and the *colmena de tierra* (*G. acapulconis*) (MI: 45%) (Figure 2). Although all species of stingless bees local experts draw them the same products (wax, honey and pollen), certain species have greater aptitude for certain products. When asked about use of honey the *colmena real* (*M. fasciata*) and *abeja zopilota* (*F. nigra*) had the highest preference. However, when we asked about use of beeswax, the *colmena de tierra* (*G. acapulconis*) and *abeja bermeja* (*S. hellwegeri*) has the highest preference, since according to local people this species produces the highest amount of beewax. Nearly 15% of people interviewed said that honey of the *abeja zopilota* (*F. nigra*) is a good remedy for treating biliary problems and diabetes.

### Management techniques

Local experts referred that in the past there were persons who went to the forest exclusively to *colmenear* (looking for bee nests), and this activity was complemented by identifying nests when carrying out other activities of their daily life. Although there is a deep knowledge on stingless bees in the study area, no raising practices or meliponiculture was recorded, therefore obtaining products derived from stingless bees is by direct extraction and partial or total destruction of nests. Currently, the use of stingless bees’ products is only occasional and may be considered in disuse. The extraction and use of stingless bees’ products was popular the last century until the 60’s where 90% of local experts identified the beginning of the decline of stingless bees’ populations, and became particularly strong in early 90’s when some species disappeared. The main causes identified as factors diminishing of stingless bees according to 75% of local experts have been the increase in the abundance of *Apis mellifera*; extractive practices and land use change (65%); drought (50%); use of insecticides, pesticides and agrochemicals (15%), and fires associated with long periods of drought (5%). Nearly 70% of local experts interviewed considered that stingless bees will disappear relatively soon.

**Figure 3:**
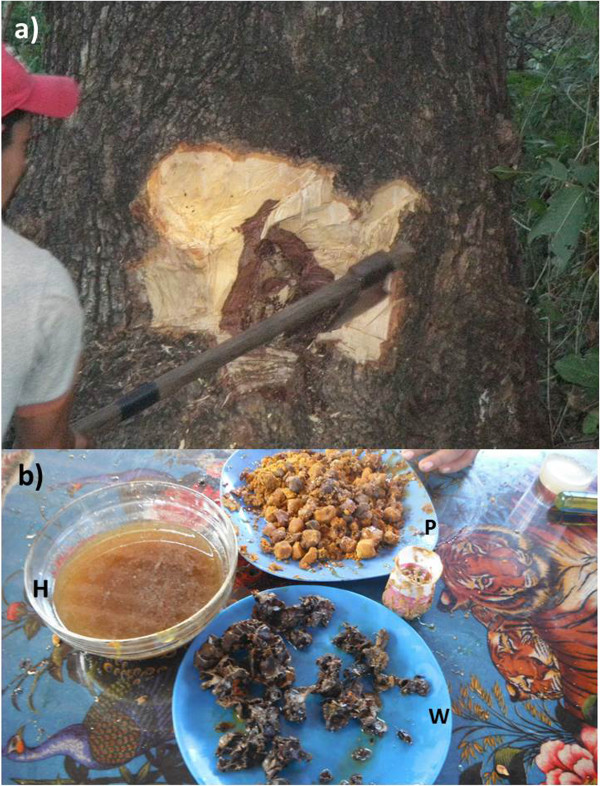
**Local extractive practices for obtaining stingless bees’ products. a)** extraction with ax; looking for the nest; **b)** stingless Bees’ products: honey (H), wax (W) and pollen – *pasacuareta* (P) Photographs: Alejandro Reyes-González.

**Figure 4:**
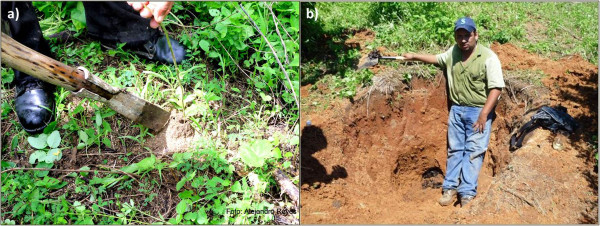
**Local extractive practices of the stingless bees that nest in the ground ( *Geotrigona acapulconis *). a)** entrance to the nest with the tool used for digging (*chuz*), **b)** nest located 1.5 m depth in relation to the entrance. Photographs: Alejandro Reyes-González.

Extractive practices of bee products are usually conducted when people need them, or during the extraction season in September and October, when availability of flower resources for bees is the highest of the year and when the nests are well provided of resources. For extracting bee products, people utilize simple tools such as axes for cutting pieces of trunks or branches in order to have access to the nests (Figure 3). Sometimes people cut the entire tree for using the bee nests and they make use of the wood. However, nearly 45% of local experts interviewed said to more frequently make use of machines like power saws. For the *colmenas de tierra* (ground colmenas), people excavate holes using tools like picks, shovels, drills. People carefully try to find the nest from its entrance emerging up on the ground towards the main body of the nest down underground (Figure 4). Gathering bee resources in this form requires fine knowledge of bees since the main body of the nest may be located on average 1.5 m deep in several directions with respect to the entrance.

**Figure 5:**
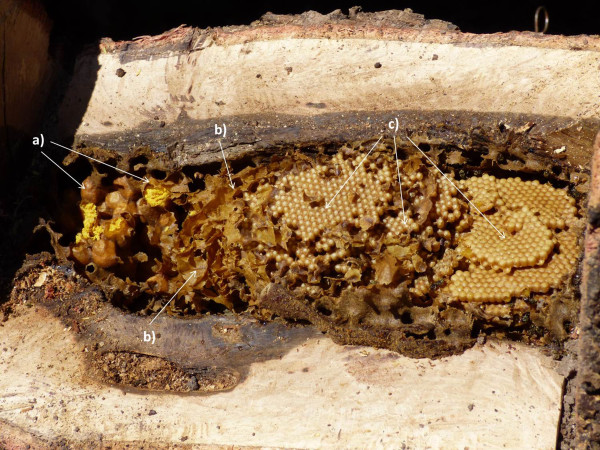
**Internal structures of a nest: a) storing pots of honey and pollen ( *tarros o tarritos *), b) wax ( *cera *) and c) brood crombs (*enjambre, mazorca de huevera *or *agrios*).** Photographs: Alejandro Reyes-González.

A nomenclature system was found for the parts of the nests. The storing pots of honey and pollen are called *tarritos* or *tarros*; the brood combs are called *enjambre*, *mazorca de huevera* or *agrios* (Figure 5). The queen bees are called *abejón*, and the wax structures (the batumen which is composed by a mixture of wax, mud and pieces of plant material, and the hive involucre) are all called *cera* (wax).

Nearly 30% of the local experts interviewed said that they extract only the honey storing pots and a bit of wax, leaving intact the cells hive re-covering then the nest allowing the bees the reconstruction of the nest until the following harvest season. However, they also said that generally after extracting products the hives become lost because of the exposition to predators, parasites, and other environmental factors. People identified as the main predators of bee hives the badger *tejón* (*Nasua narica*), *tlacuache* (*Didelphis marsupialis*), skunk *zorrillo* (*Mephitis* sp), coyote (*Canis latrans*), armadillo (*Dasypus novemcinctus*), ants and the wasps locally called *avispas carneras*. Two persons recognized the parasitic interaction of the stingless bee *limoncilla* (*L. chamelensis*) with other stingless bee species, as well as with the euorpean bee (*Apis mellifera*).

On average, every local expert or *colmenero* extracted four hives of stingless bees per year, obtaining on average 6 L of honey and 4 Kg of beewax; however, some local experts said to collect up 10 to 12 hives per year obtaining on average 18 L of honey and 24 Kg of wax, amounts that indicate that the activity and use of these resources were important for people of Nocupétaro. Even when the *colmeneros* extract a relatively high amount of stingless bee’s products, commercialization is restricted to wax and it occurs at low scale, as candles in small store or for grafting. Honey is collected for direct consumption of households that collect it.

## Discussion

According to Ayala [2], have been described 11 genera and 46 species of stingless bees for Mexico, and particularly for the state of Michoacan have been described 8 genera (72% of the total) and 13 species (28% of the total). In the study area we recorded a relatively high species richness of stingless bees including 7 genera and 8 species (representing 87.5% and 61.5% of the species recorded in Michoacan). It is important to notice and highlight that in this study *L. chamelensis* was identified as a new record of species for the Balsas River Basin of Michoacán, and was recorded the presence of *M. fasciata*, which is endemic to the region and are recognized as mountain species, inhabiting zones of relatively higher elevations [2,3]. The new record of *L. chamelensis* for the area is transcendent since its presence depends in turn on the abundance of other stingless bees’ species because it is a clepto-biological species.

An important aspect derived from this study is that it is possible to suppose that the number of stingless bees’ species in the study area is higher because the registration of the *abeja pintilla*, *abeja prieta esculcona* and the *abeja sapito*. This kind of stingless bees where mentioned by local experts but not collected and they could represent different. Additionally we can assume this because were not explored all vegetation types, particularly crops such as sesame or peanut.

Local identification of stingless bees’ species incorporates not only morphological but also ecological, behavioral, and social characteristics, as described in other studies and cultural contexts [33].

There are recently published works that integrate exhaustively the status of meliponiculture on a global scale [12], which underscores the cultural importance of this activity. Instead, this paper focuses on a much more basic activity which is the management of wild populations of these bees. In our study, all the species described are used and there were no related meliponiculture management practices rather extractive practices. This condition had already noticed by Hendrichs [14], who states that in the Balsas region were well known the *colmeneros*, who practiced a specialized activity, but mainly by the extraction process.

Doing an exhaustive literature review, we found the report of 24 species of stingless bees that are used in Mexico in some form. These species are: *Cephalotrigona eburneiventer* (Schwarz, 1948) [3], *Cephalotrigona oaxacana* (Ayala, 1999) [3], *Cephalotrigona zexmeniae* (Cockerell, 1912) [3,34], *Frieseomelitta nigra* (Lepeletier, 1836) [22], *Geotrigona acapulconis* (Strand, 1919) [3], *Melipona beecheii* (Bennett, 1831) [3,10,18,19,15,22], *Melipona colimana* (Ayala, 1999) [3], *Melipona fasciata* (Latreille, 1811) [3,15,21], *Melipona lupitae* (Ayala, 1999) [3], *Melipona solani* (Cockerell, 1912) [20,23], *Melipona yucatanica* (Camargo, Moure, Roubik, 1988) [22], *Nannotrigona perilampoides* (Cresson, 1878) [3,13,20,34,35], *Paratrigona guatemalensis* (Schwarz, 1938) [3], *Partamona bilineata* (Say, 1837) [3], *Plebeia frontalis* (Friese, 1911) [34], *Plebeia manantlensis* (Ayala, 1999) [3], *Plebeia moureana* (Ayala, 1999) [22], *Plebeia pulchra* (Ayala, 1999) [34], *Scaptotrigona mexicana* (Guérin, 1845) [3,10,19,20,35], *Scaptotrigona pectoralis* (Dalla Torre, 1896) [3,20,34,35], *Scaptotrigona hellwegeri* (Friese, 1900) [21], *Tetragonisca angustula* (Lepeletier, 1811) [3,20,35], *Trigona nigérrima* (Cresson, 1878) [3], *Trigonisca maya* (Ayala, 1999) [34]. In this sense, our research incorporates two new useful species, *Trigonisca* sp., (the recorded use of honey is medicinal) and *L. chamelensis*, (the recorded use of honey is medicinal and people recognize that the ingestion of this species’ honey, and pollen, cause *miserere*) totaling 26 useful species. This represents that of the 46 species described for Mexico [2,3], 56.5% are useful. However, we consider of great importance join efforts to integrate a national inventory of stingless bees.

On the other hand it is clear that there are stingless bees’ species with a higher level of importance because the local preference of their products. This differential valuation in specific cultural contexts has been described in other studies, mainly in plants [32]. In this case, species widely locally known such as *F. nigra, S. hellwegeri, M. fasciata and G. acapulconis* at the same time are most preferred species for use. This represents a universe of species with high potential for local management and technological innovation in the study area. For this purpose would be of great importance to develop ecological studies to determine the distribution and abundance of stingless bees. This information is essential to generate plans for sustainable management of this group of insects. In this sense, it is important to note that *M. fasciata* seems to be a rare species since we collected only two specimens in one site, and no nests were identified, and *G. acapulconis* nests on the ground making it difficult to use.

While it is true that honey is one of the main stingless bees’ products, is also true that there is a need to establish quality criteria. Recently the demand for natural, organic and homeopathic products has increased, including those of stingless bees and particularly for the stingless bees’ honey have not yet been defined formal norms or quality standards [36], which is quite complex because of the variety of species and types of honey specific to each region [37]. Thinking about the possibility of promoting alternative management projects that represent economic benefits, characterize the physicochemical properties of stingless bees’ honey represents a relevant field of research that has to be developed in the study area.

During the fieldwork we recorded general information about eight different types of Hymenoptera that people of Nocupétaro know such as *Xylocopini* (*xicota*), *Bombini* (*abejorro, guaricho, guaricho grande*) and *Vespidae* (*avispa arápara o ahogadora, avispa carnera,* a*vispa guitarrilla, huevo de toro*). This is relevant since other authors [38] highlight the cultural importance of the wasp *uauapu* (*Polibya occidentalis* Buysson, 1905) between Purépecha culture (territory adjacent to the study area). This motivates the exploration of the relationship between society and insects in the study area, including stingless bees but also in a broader sense, including other insects.

## Conclusions

Bees are among the main pollinators of flowering plants throughout the world and their conservation is a current main issue of both science and society. This is a general premise for all bees’ species but it acquires particular relevance for those species that in addition to pollination represent provision ecosystem services as are several species of *Meliponini*. Nonetheless local experts of Nocupétaro perceive and recognize that extraction rates of bee products have decreased more recently alongside the increase of vegetation clearing and use of insecticides threatening the permanence of stingless bees, local knowledge and the use of resources they provide persist in the study area. For this, it is relevant to establish the consequences that environmental problems may have for both, populations of stingless bees and local culture associated with them. In this sense ethnozoological and ethnoentomolgical studies are a key reference to direct these efforts.

The information generated suggests that conservation associated to use of bees (integral meliponiculture) is still possible to be enhanced in Nocupétaro. Mainly because the resource and local knowledge remain in the study area. Also, for the interest of the *colmeneros* to develop this activity. No forms of cultivation management was recorded in the studied area, but important experiences in other regions of Mexico offer the possibilities to encourage efforts for recovering the local techniques innovating both knowledge and management practices based on those from other areas of Mexico.

## Consent

Written informed consent was obtained for the publication of this report and any accompanying images.

## Competing interests

The authors declare that they have no competing interests.

## Authors’ contributions

The main author ARG was responsible for literature review, research design, fieldwork, systematization and analysis of field data, wrote the first draft and concluded the final version of this paper. ACG main coordinator-supervisor of the research project contributed with the research design, participated in fieldwork, systematization and analysis of data and reviewed several drafts of the manuscript. ORS contributed with the research design, review of background literature and the discussion of theoretical aspects of the study. AA contributed to the review of background literature and the discussion of theoretical aspects of the study. AC contributed to designing and following progress of the research, field work and data analyses, and made substantial contributions to the manuscript. All authors have read and approved the final manuscript.
